# Assessment of bias in carbon isotope composition of organic leaf matter due to pre‐analysis milling methods

**DOI:** 10.1002/rcm.9134

**Published:** 2021-06-21

**Authors:** Savannah Worne, Jack H. Lacey, Cameron Barr, Cameron Schulz, Melanie J. Leng

**Affiliations:** ^1^ British Geological Survey National Environmental Isotope Facility Keyworth Nottingham NG12 5GG UK; ^2^ Department of Geography, Environment and Population University of Adelaide Adelaide SA 5005 Australia; ^3^ Department of Environment and Science Queensland Government Brisbane City Queensland 4000 Australia; ^4^ School of Biosciences University of Nottingham Loughborough LE12 5RD UK

## Abstract

**Rationale:**

Stable isotope analysis of leaf material has many applications including assessment of plant water‐use efficiency and palaeoclimatology. To facilitate interpretations of small shifts in the carbon isotope composition (δ^13^C) of leaves, accurate and repeatable results are required. Pre‐sample homogenisation is essential to ensure a representative sample is analysed, but can also introduce error.

**Methods:**

We investigate how different grinding methods (freezer‐milling and ball‐milling) affect the carbon content and δ^13^C of tree leaves from a wetland in Queensland, Australia, commenting on how increased temperature, sample contamination, sample loss or poor homogenisation may impact results.

**Results:**

No alteration of leaf δ^13^C is observed due to different milling methods, although there may be a significant increase in %C of samples processed using ball‐milling.

**Conclusions:**

We suggest %C variability is possibly due to contamination from abraded plastic vials or insufficient homogenisation during ball‐milling, with no significant impact on δ^13^C. Overall, we suggest that intermittent ball‐milling may be the best solution to reduce costs, preparation time and use of liquid nitrogen, aiming to achieve complete homogenisation using the shortest possible duration of milling.

## 1 INTRODUCTION

Stable isotope analysis of leaf matter is commonly undertaken in a range of environmental and biological research fields, where the carbon isotope composition (δ^13^C) of a leaf is primarily determined by fractionation of carbon dioxide (CO_2_) during uptake.^1^, ^2^ The δ^13^C of a leaf is therefore a reflection of gas exchange and chemical processes associated with plant photosynthesis and respiration.^1^, ^2^, ^3^, ^4^, ^5^ More specifically, δ^13^C of leaf matter is dependent on a range of biological pathways including CO_2_ assimilation,^2^ stomatal limitations during photosynthetic activity,^2^, ^6^ leaf metabolism^7^ and post‐photosynthetic fractionation including Rubisco carboxylation.^3^, ^8^ This information, sometimes in combination with other stable isotope analyses (primarily oxygen and nitrogen isotopes), is then used to better understand the relationship between water use and transpiration efficiency in all plant types (C3 or C4 plants)^1^, ^9^, ^10^, ^11^, ^12^, ^13^ and can be used to optimise genotypes for crop breeding.^9^ δ^13^C can also be used to reconstruct palaeoclimatic conditions, where the preserved δ^13^C measures of fossil remains are a reflection of the atmospheric CO_2_ supply and environmental conditions during the lifespan of the leaf.^14^, ^15^ As the range of δ^13^C values in leaf material as a result of environmental or physiological variability can be relatively small (*ca* 4‰ range for C4 plants),^1^, ^10^, ^16^ it is essential that reliable and accurate stable isotope measurements are made.

Analytical error can be introduced in a number of steps during stable isotope analysis. While the analytical precision from mass spectrometry is typically <0.1‰ with sample repeatability <1*σ*,^17^ a much larger proportion of the error in isotope values may occur as a result of the sample preparation process. Unlike in other environmental sample types, leaf material does not typically require chemical pre‐treatment as it is almost entirely organic in composition; therefore the most notable introduction of error is likely to occur during homogenisation of samples.^18^ It has been shown that carbon and nitrogen content (%C and %N, respectively), as well as the δ^13^C and nitrogen isotope (δ^15^N) compositions, are highly variable within different density fractions of soil materials, which is related to degradation processes.^19^, ^20^ Similarly, for δ^15^N analysis, analytical precision is increased by fine grinding of soil and plant materials (<0.053 mm).^21^, ^22^, ^23^, ^24^, ^25^ It is also important to homogenise whole‐leaf samples to attain an accurate representation of total leaf carbon, as δ^13^C can vary between the bottom and top of the leaf sample; for example, an intra‐leaf variability of *ca* 1‰ was found in fossil leaves from Gujarat, western India.^26^ Therefore to overcome variable allocation of carbon within a leaf,^26^, ^27^, ^28^ as well as variable alteration of organic carbon during leaf senescence,^13^, ^29^ samples must be homogenised to ensure that a representative sample is analysed.

Routinely used homogenisation methods often employ milling of samples into a fine powder, which can then be weighed out for micro‐analysis.^30^ Although there are a range of methods used to homogenise leaf tissues, it is imperative that the stable isotope composition of the organic compounds is not altered during this process. Common methods of milling samples often include mechanical grinding, through either the use of a ball‐mill or cryogenic milling techniques, where the latter include either grinding by hand in a pestle and mortar in the presence of liquid nitrogen or using a mechanical freezer mill.

Mechanical freezer‐milling grinds a sample using a solenoid that is oscillated back and forth inside a metal vial. This method provides better homogenisation relative to ball‐milling or hand‐grinding, and reduces the likelihood of loss of volatile organic material (as CO_2_) and the generation of black carbon^18^ as the samples are ground in a bath of liquid nitrogen at *ca* −196°C. However, this method can be time‐consuming and expensive and only a small number of samples can be ground at the same time. Furthermore, use of liquid nitrogen also has specific safety and staff training requirements.

Conversely, ball‐milling can process multiple samples in a short period of time and does not require the use of liquid nitrogen. However, this process may introduce heat to the samples through friction with the ball bearings as the sample is pulverised. The heat that builds up will be dependent on the duration of the milling and the quantity and material of the ball bearings used (the larger the number or density of ball bearings, the greater the friction).^31^, ^32^ Although a previous study which assessed the alteration during hydrothermal decay of fossil plant samples showed there was no isotopic change before samples reached 200°C,^33^ lower temperatures are routinely used for drying samples in an oven (<40°C). This minimises the loss of volatile components prior to analysis, which may alter the δ^13^C value of the organic carbon fraction.^18^, ^34^ To overcome heating during ball‐milling, intermittent milling can be used to allow the samples to cool in between milling periods to help prevent or reduce potential isotope fractionation.^31^

To date, there have been no investigations of the impact of pre‐analysis sample homogenisation on leaf carbon, specifically. A similar study of the homogenisation of wood samples for tree‐ring analysis using ball‐milling,^31^ which varied the duration of milling, the number of ball bearings and also the impact of intermittent milling, demonstrated no significant δ^13^C alteration, indicating that any heat development during ball‐milling had no distinguishable effect on isotope values.^31^ Although this study did not report the temperature that samples reached as a result of ball‐milling, typically mixer ball mills have been shown to increase temperatures up to 50°C after 1 h of milling in a SPEX mixer mill,^35^ although higher temperatures of up to 66°C have also been reported.^36^ This range of temperatures likely results from different milling ball materials, densities and sizes, as well as the material of the mill itself.^32^ On the other hand, ball‐milled soils showed *ca* 8% higher total C compared to a gentler roller mill method. This was attributed to abrasion of the ball bearings and grinding vessel, although the resultant impact on isotope values was not assessed.^30^ Similar studies that assessed δ^13^C alteration in cellulose and wood samples that were homogenised using freezer‐milling showed negligible difference with the initial sample and within the processed sample.^37^

Aside from temperature concerns, other issues that result from these pre‐analysis sample homogenisation methods include sample contamination, sample loss and cost. For example, a δ^13^C bias of around 7‰ was found in ball‐milled wood samples due to contamination from polypropylene plastic vials.^38^ Furthermore, *ca* 28% sample loss was reported following freezer‐milling of cellulose samples, as well as excessive time and cost associated with the labour‐intensive cleaning of the freezer mill using compressed air.^37^ Indeed, the cost of a freezer‐mill in itself is high, costing up to approximately £20 000, as well as substantial operating costs due to the infrastructure requirements and the replenishment of liquid nitrogen. In comparison, the ball‐mill used in the experiments reported here (Retsch MM400) cost approximately £8 500, and has significantly lower running costs as a larger number of samples can be analysed per run.

Overall, it is essential to evaluate the impact of pre‐analysis sample homogenisation using milling on the stable isotope composition of organic materials to facilitate the continued use of these analyses in important socio‐economic research fields including agricultural and environmental sciences. This is particularly relevant for the continued development of the δ^13^C proxy in leaves, which can provide essential information on water stress and water‐use efficiency in regions that are vulnerable to drought. Therefore, the study reported here aimed to quantify the variable impact of ball‐milling and freezer‐milling on the δ^13^C of leaf samples in order to provide recommendations for future analysis of leaf sample material.

## 2 METHODS

### 2.1 Leaf sample material

**FIGURE 1:**
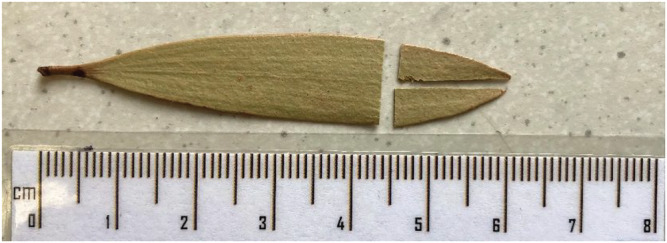
Example of a leaf sample analysed in this study. Leaf tips were cut, with half of the sample homogenised by freezer‐milling and the other half by ball‐milling

Leaves of the tree species *Melaleuca quinquenervia*, a broad‐leaved paperbark (family Myrtaceae), were collected from Swallow Lagoon on Minjerribah (North Stradbroke Island) (27.499° S, 153.455° E).^16^ Fragments of *M. quinquenervia* have been preserved in wetland sediments at this location and have been used in previous studies to investigate the palaeoclimate of the region and elucidate the relationship between climate and leaf δ^13^C in subtropical environments.^39^ Leaves were collected from the same branch of the same tree at roughly monthly intervals over several years. The leaves were refrigerated immediately after collection before being freeze‐dried to preserve the organic fraction and prevent degradation. The data presented here are from 40 samples taken from this archive. For each sample, the tips (10–15 mm) of the five smallest leaves within each collection were cut and split in half, with half of each leaf sample to be homogenised by freezer‐milling and the other half to be processed by ball‐milling (Figure 1). In this way, each homogenised sample analysed was a composite of five leaves from each collection. Leaves were sampled in this manner with the aim of gaining a representative sample of the most recent leaves, and avoid inter‐leaf variation dominating the carbon isotope composition.

### 2.2 Homogenisation using ball‐milling

The cut leaf tips were broken up and placed in 2 mL polypropylene tubes with three stainless steel ball bearings (approximately 4 mm in size). The tubes were then placed into a Retsch MM400 ball‐mill ‘rack’ (capacity of 48 samples) that was shaken back and forth at a frequency of 30 Hz for 20 s. Samples were then left to rest for 5 min and this was repeated five times (Table S1, supporting information). The 5 min rest interval was used to keep frictional temperatures low; this method is henceforth termed ‘intermittent ball‐milling’. To investigate how the higher temperatures caused by more aggressive ball‐milling would affect δ^13^C, a subsample of the homogenised leaf samples was re‐milled an additional five times at a frequency of 30 Hz for 45 s, with only 1 min rests between milling intervals (Table S1, supporting information). This latter method is henceforth termed ‘ball‐milling’.

### 2.3 Homogenisation using freezer‐milling

Prior to sample homogenisation, all milling equipment was washed thoroughly with deionised water before being dried in a sterile crucible. Leaf tip samples were broken up and transferred into stainless steel tubes (diameter 8 mm, length 51 mm). A stainless steel rod (diameter 4 mm, length 18 mm) was placed into each tube and the ends were then sealed with stainless steel caps. Three metal tubes were placed inside a larger polypropylene tube (diameter 21 mm, length 91 mm), which was capped at both ends and positioned inside a SPEX CertiPrep 6850 freezer‐mill, which had been cooled in a bath of liquid nitrogen at −196°C. Four polypropylene tubes can be housed in the freezer‐mill (12 samples per run) and each tube casing has several holes to aid flow of liquid nitrogen to cool the sample material. The metal rods were oscillated at a speed of 10 impactor movements per second for 2 min Table (S1, supporting information). Once complete, samples were left to warm to room temperature before being removed from the tubes and transferred to vials.

### 2.4 Stable isotope analysis

Around 0.8 mg of each homogenised sample was weighed directly into Sn capsules (8 mm ×5 mm; Sercon Ltd) using a microbalance (Sartorius Cubis® II), with their weight recorded to the 0.001 mg level, and transferred to a multicell sample tray. Analysis was undertaken using an Elementar Vario ISOTOPE cube elemental analyser coupled to an Isoprime precisION isotope ratio mass spectrometer with an onboard centrION continuous flow interface system. The sample isotope ^13^C/^12^C ratio is reported in delta (δ) notation in per mill (‰) and was calibrated to the VPDB international reference scale using a multipoint calibration against USGS24 (−16.0‰), USGS61 (−35.0‰) and a laboratory working standard BROC3 (−27.6‰). The working reference material BROC3 has been calibrated for δ^13^C using IAEA‐CH‐6 (−10.4‰), USGS54 (−24.4‰), USGS40 (−26.4‰) and B2174 (urea, Elemental Microanalysis Ltd; −36.5‰). BROC3 (41.3%C and 4.9%N) was also used to calculate the carbon and nitrogen elemental content of samples. External precision (1*σ*) was <0.05‰ for δ^13^C based on replicate analyses of the reference materials. Given the lower %N of sample, there was not enough material to analyse δ^15^N. Also, given that previous studies found there was no significant difference in δ^15^N or %N due to milling technique of soils,^30^ repeat analyses for δ^13^C (which require considerably less material) were prioritised for this study. All samples processed by each method (120 in total) were run in duplicate (240 data points), with outlier samples and samples exhibiting high variability (*ca* 10% of samples) being run in triplicate, to create a total of 270 data points. Repeatability for sample material is discussed below.

## 3 RESULTS AND DISCUSSION

### 3.1 %C and %N of leaf matter

The average %C of all leaf samples was 53.27% (±0.55%), ranging from 43.31% to 62.85%. The average %C of freezer‐milled samples was slightly lower, but within error, at 52.03% (±0.48%), while the %C was higher for both intermittent and ball‐milling methods (53.63 ± 0.60% and 54.12 ± 0.58%, respectively) (Table 1). Higher %C is observed with increasing duration of ball‐milling. This suggests that the method of homogenisation may cause a shift in the %C of the leaf, with ball‐milling producing an increase of *ca* 2% relative to freezer‐milling, as well as increasing %C with duration of ball‐milling. Results here show that the %N did not follow the trend observed in %C, with similar %N occurring in intermittently ball‐milled (0.66%), freezer‐milled (0.64%) and continuously ball‐milled (0.56%) samples. Although the %N is too low to accurately assess the impact of milling using the small sample sizes analysed in this study (*ca* 0.8 mg per sample), this result is in line with previous studies which suggest that N concentrations are not affected by particle size^20^ or by the method of milling.^30^

**TABLE 1:** Average %C and %N of 270 samples from 40 collections, categorised by method of homogenisation

Method	Average %C	±1*σ* of duplicates/triplicates	Average %N	±1*σ* of duplicates/triplicates
Ball‐milling	54.12	0.58	0.56	0.05
Intermittent ball‐milling	53.63	0.60	0.66	0.07
Freezer‐milling	52.03	0.48	0.64	0.06

Previous studies found that grinding soils using mechanical ball‐milling caused an average increase of 8% in total C content compared to gentler methods, with an additional 3.5% total C content found after re‐milling, attributed to abrasion of steel ball bearings.^30^ In a similar study, the contamination of samples by abraded metal was also identified as a potential cause in altering the concentration of a range of elements in leaf and stem material during grinding, in particular the finer fraction of the homogenised sample.^20^ This contamination could have occurred either from microparticles remnant in the abraded metal from previously homogenised samples or from the metal itself. However, it was also acknowledged by both studies that uneven distribution of elements in the original plant tissue and variable levels of homogenisation could also have caused the different elemental composition of different size fractions.^20^, ^30^ The larger particles that are more resistant to crushing are also more likely to contain higher organic concentrations.^30^ In this study, although five leaves were combined for each collection, there is still potential inter‐leaf variability within a sample collection, which may have caused a range of values to be preserved within a homogenised sample.

Alternatively, contamination of samples from the polypropylene tubes used during ball‐milling is also a potential cause of change in %C.^38^ To quantify the potential contamination, shavings of a 2 mL polypropylene transport tube used for ball‐milling were analysed and returned a %C of *ca* 84.06 ± 0.07% (*n* = 3). To cause the 2.09% increase in %C observed in continuously ball‐milled samples (compared to freezer‐milled samples which were processed in stainless steel vessels), this would require 6.5% of the measured sample to be from plastic contamination. Given that samples were weighed at *ca* 0.8 mg, this corresponds to 0.05 mg of polypropylene. Decreased %N in the continuously ball‐milled samples gives some support for potential plastic contamination, as polypropylene contains no nitrogen. Although it seems very unlikely that such a significant proportion of the sample would result from abrasion of the polypropylene during ball‐milling, plastic contamination may be, at least in part, responsible for the shift in %C.

### 3.2 δ^13^C of leaf matter

**FIGURE 2:**
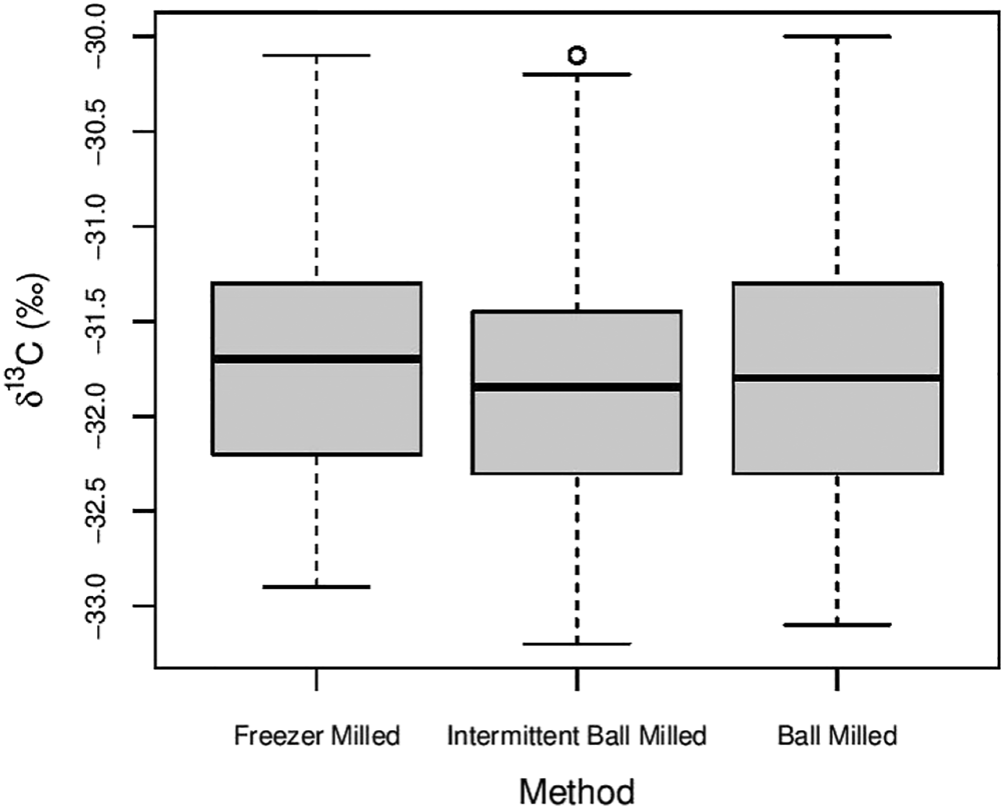
Boxplots of δ^13^C for 270 leaf samples analysed from 40 leaf collections, categorised by method of homogenisation. The open circles represent outliers which are 1.5 times outside the interquartile range

The average δ^13^C of all leaf samples analysed including duplicates and triplicates was −31.78‰ (*n* = 270), with similar average values and standard deviations for each method of homogenisation (Table 2). The range and distribution of values were also similar (Figure 2), with one‐way ANOVA undertaken using R statistical computing software^40^ indicating there was no significant difference in the variability of the data for each milling method (*f* = 0.73, *p* > 0.05).

**TABLE 2:** Average δ^13^C of 270 samples analysed from 40 leaf collections, categorised by method of homogenisation

Method	Average δ^13^C (‰)	*σ* of duplicates/triplicates (‰)
Ball‐milling	−31.78	0.04
Intermittent ball‐milling	−31.82	0.06
Freezer‐milling	−31.73	0.04

As the %C data indicated potential contamination of samples from the abrasion of the polypropylene tubes during ball‐milling, the potential impact on δ^13^C was also assessed. The δ^13^C of the tubes used in this experiment was measured to be −27.68 ± 0.06‰ (*n* = 3). Using the previously calculated contamination of 6.5%, a +0.26‰ shift should have occurred in the ball‐milled samples compared to freezer‐milled ones. However, results here suggest that the δ^13^C of leaf material is not affected by the method of homogenisation, where both freezer‐milling and ball‐milling produce average δ^13^C values that are not statistically different. Furthermore, the repeatability of the data is excellent, with an average standard deviation of 0.05‰ across duplicate and triplicate data. Similarly, *t*‐test analysis of averaged duplicate/triplicate data also revealed that there was no significant difference in δ^13^C between any of the methods of homogenisation (Table 3). Given that it is extremely unlikely that such a large amount of plastic would be abraded from the vials during ball‐milling, and that there was not a consistent change to higher δ^13^C with increased duration of ball‐milling, we suggest that it is unlikely that polypropylene derived from vial abrasion during ball‐milling is the main cause of observed %C. Further work to quantify the exact quantity of polypropylene abraded from vials that enters the sample during ball‐milling, as well as assessing the impact on results, is required.

**TABLE 3:** Results of *t*‐tests using averaged duplicate/triplicate δ^13^C data for 40 leaf collections, categorised by method of homogenisation

Welch two‐sample *t*‐test	*t*	*p*	99% confidence
Freezer‐milling versus intermittent ball‐milling	0.77	0.44	Not significantly different
Intermittent ball‐milling versus ball‐milling	−0.37	0.71	Not significantly different
Freezer‐milling versus ball‐milling	0.40	0.69	Not significantly different

## 4 CONCLUSIONS

Stable isotope analysis of leaves is a commonly used proxy which can be applied in a multitude of research fields; however, application of δ^13^C leaf data is reliant on accurate and repeatable results to facilitate interpretation of small shifts in isotope values. Our results show that, although there may be a slight increase in %C as a result of ball‐milling during pre‐analysis homogenisation, the δ^13^C is unaffected by choice of grinding method. There are various explanations for why ball‐milling may have caused an increase in %C, including sample contamination as a result of abrasion of the polypropylene plastic vials and metal ball bearings, insufficient homogenisation where larger particles are likely to have higher organic C content or variable carbon storage between and within leaves from the same collections that were homogenised into one sample. Overall, although freezer‐milling techniques are often considered the best method for processing plant samples for δ^13^C analysis, as they do not introduce heat/friction to the pre‐analysis, we show that homogenisation using ball‐milling can be equally as effective and does not influence the δ^13^C results. Given that ball‐milling is a cheaper and quicker process, and also reduces human exposure to chemicals hazardous to health, we suggest that ball‐milling is a suitable alternative to freezer‐milling for analysing samples for δ^13^C. Given the observed shift in %C with increased duration of ball‐milling, we suggest that intermittent or continuous ball‐milling be used for the shortest possible duration, to somewhat reduce potential influence on %C. However, using any method, it is of critical importance that complete homogenisation is achieved to ensure representative and accurate results are produced.

### PEER REVIEW

The peer review history for this article is available at https://publons.com/publon/10.1002/rcm.9134.

## Supporting information


**Table S1.** Methodological details for each type of homogenisation

**Data S1**. Milling methods δ^13^C data

## Data Availability

All data presented in this paper are available in the supporting information.
