# Supplementary material for: Assessment of bias in carbon isotope composition of organic leaf matter due to pre‐analysis milling methods
Source: Rapid Commun Mass Spectrom. 2021 Jun 21;39(Suppl 1):e9134. doi: 10.1002/rcm.9134 (PMC12062769; doi:10.1002/rcm.9134)
Supplement: Supplementary file 1 — Table S1. Methodological details for each type of homogenisation [file RCM-39-e9134-s001.docx]

Supplementary Table 1) Methodological details for each type of homogenisation

| Method | Machine | Speed | Time Milled (sec) | Rest Interval (min) | Repetitions |
| --- | --- | --- | --- | --- | --- |
| Ball-milling | Retsch MM400 ball-mill | 30 Hz | 45 | 1 | 5 |
| Intermittent Ball-milling | Retsch MM400 ball-mill | 30 Hz | 20 | 5 | 5 |
| Freezer-milling | SPEX CertiPrep 6850 freezer-mill | 10 impactor movements/sec | 120 | N/A | 0 |
